# Psychometric Properties of the Attitudes to Ageing Questionnaire—Short Form (AAQ-SF) in Sri Lanka

**DOI:** 10.3390/ijerph23030383

**Published:** 2026-03-17

**Authors:** Himalshi P. S. Kristoper, Lidia Suárez, Nigel V. Marsh

**Affiliations:** 1 Kalana Mithuru Sevana, University of Kelaniya, Kelaniya 11600, Sri Lanka; himalshipoornimaserasinghe.kristoper@my.jcu.edu.au; 2 School of Social and Health Sciences, James Cook University, 149 Sims Drive, Singapore 387380, Singapore; lidia.suarezabalos@my.jcu.edu.au; 3 Tropical Futures Institute, James Cook University, 149 Sims Drive, Singapore 387380, Singapore; 4 Margaret Roderick Centre for Mental Health Research, James Cook University, 149 Sims Drive, Singapore 387380, Singapore

**Keywords:** older adults, attitudes toward aging, AAQ-SF, Sri Lanka, psychometric properties, retirement homes

## Abstract

**Highlights:**

**Public health relevance—How does this work relate to a public health issue?**

- There is a gradual shift away from aging at home (aging in place), with more older adults considering alternative living arrangements in later life.
- It is increasingly important for researchers to equip policymakers and government agencies with the essential tools to allow them to optimize the living environment for older adults, and their quality of life.

**Public health significance—Why is this work of significance to public health?**

- This study adds to research supporting the value of assessing attitudes to aging in adults in their 50s and those living in Asia.
- By examining the validity of this measure in a developing country, this manuscript contributes additional evidence for its usefulness and the value of the constructs it assesses.

**Public health implications—What are the key implications or messages for practitioners, policymakers and/or researchers in public health?**

- Attitude to aging is a multifaceted construct and its assessment requires consideration of all the different aspects.
- Attitudes to aging are an important contributor to overall quality of life in older adults.

**Abstract:**

In Sri Lanka, despite cultural norms traditionally encouraging older adults to live with their families, a growing number now reside in homes for the elderly. Limited research has explored how attitudes toward aging affect these institutionalized older adults. This study examined the construct validity of the 12-item Attitudes to Ageing Questionnaire—Short Form (AAQ-SF) and its association with quality of life among 317 residents of 13 retirement homes. The AAQ-SF showed acceptable internal consistency for the total scale (Cronbach’s α = 0.71), though subscale reliabilities were modest. Confirmatory factor analysis supported good construct validity for a three-factor model—χ^2^/df = 1.91, TLI = 0.94, CFI = 0.95, RMSEA = 0.05—but the results suggested multidimensionality of the psychological growth factor. Positive attitudes toward aging were associated with greater quality of life, providing some evidence for convergent validity. The findings suggest that the AAQ-SF may be appropriate for assessing attitudes toward aging among older adults in Sri Lanka, though further validation is recommended.

## 1. Introduction

The proportion of older adults worldwide is rising at an unprecedented pace and is projected to continue accelerating over the coming decades, while the number of children and youth has begun to decline. The number of adults aged 65 years or older is projected to double by 2050, surpassing 1.6 billion [1]. In 2023, the United Nations estimated that all less-developed countries are expected to experience significant growth in the number of older adults between 2023 and 2050. In South Asia, Sri Lanka stands out for its aging trend, with projections indicating that by 2041, one in every four persons will be an older adult [2].

While this demographic transition is unavoidable, collective actions and policy decisions can shape its impacts. Conversely, with appropriate planning, governments can address these challenges while promoting opportunities for all individuals and ensuring that no one is left behind. Population aging impacts all levels of society, extending beyond older adults and affecting areas such as healthcare, education, employment, and taxation. Experiences and policies throughout the life course shape well-being in later life [1].

In Sri Lanka, cultural traditions have long encouraged older adults (aged 55 years and above) to live with their families. Many still reside with a spouse or adult children. However, an increasing number (particularly widowed women who often assume caregiving responsibilities for grandchildren) now live alone or transition into residential care facilities [2]. The presence of living children plays an important protective role for both mental and physical health, particularly among older adults aged 60 and above, compared to those without children [3]. Parental status and age may therefore play a role in shaping older adults’ living arrangements and their access to social and emotional support in later life [3,4]. Despite the rising proportion of institutionalized older adults, research on the factors influencing their well-being (particularly mental health and quality of life) remains limited.

Quality of life among older adults is strongly linked to their subjective perceptions of aging [5]. Positive perceptions of aging are key determinants of well-being and quality of life in healthy aging. Previous research has shown that positive attitudes toward aging are negatively associated with depression, anxiety, poor health, and low self-esteem, and positively associated with quality of life [6,7]. However, attitudes toward aging cannot be understood solely as individual-level psychological constructs. They may also be shaped by broader sociocultural contexts and environmental influences, such as institutional living arrangements. Nevertheless, findings regarding the role of cultural values remain inconclusive. Some studies suggest that cultural values are not strong predictors of attitudes toward older adults. In contrast, the same line of research proposes that cultural values may better predict global evaluative judgments, whereas personal values are more directly associated with individual attitudes [8].

Moreover, attitudes toward institutional living arrangements are similarly complex. Although some older adults hold positive views of residential homes, recognizing that such settings provide opportunities for social engagement, friendship formation, safety, and participation in structured activities that promote personal growth and mastery [9,10], others perceive institutional living negatively. For some individuals, relocation to residential care may be associated with feelings of diminished dignity, reduced autonomy, loss of freedom, and a decline in sense of purpose [4]. Attitudes toward aging operate in two ways: one reflects general beliefs about aging, and the other concerns individuals’ attitudes toward their own aging [5]. Simply put, attitudes toward aging influence older adults’ experiences, understanding, and expectations of their own aging process, as well as their present and future lives in old age [5]. Therefore, evaluating attitudes toward aging and addressing older adults’ negative attitudes toward aging are essential for promoting social adaptation, and for developing effective policies and programs that support older adults’ well-being [5,11].

The present study examines the psychometric properties of a measure of attitudes toward aging among older adults residing in retirement homes in Sri Lanka. This measure is the Attitudes to Ageing Questionnaire—Short Form (AAQ-SF [6], which is the short version of the Attitudes to Ageing Questionnaire (AAQ) developed by Laidlaw [12], and it is used to assess older adults’ attitudes towards the process of aging. The original AAQ contained 24 items, and the short form (i.e., the AAQ-SF) is made up of 12 items. Both measures comprise three factors: perceptions of psychological growth (PG), physical change (PC), and psychosocial loss (PL). PG represents the accumulation of personal wisdom that emerges through aging, PC refers to individuals’ perceptions of the physical and health-related alterations accompanying the aging process, and PL denotes the negative appraisal of aging, which in later life is conceptualized as a stage marked by psychological and social decline.

The AAQ-SF has been validated in different countries with community-dwelling older adults, and the findings are relatively consistent regarding its factor structure (Table 1). Overall, the construct of attitudes to aging, as measured by the AAQ-SF, is summarized as a three-factor construct made of PG, PC, and PL.

As shown in Table 1, the internal reliability of the PG scale was the lowest compared to PC and PL. This has been explained by the multidimensionality of the PG scale, which seems to measure the benefits of growing old towards the self (items 1 and 2) and others (items 8 and 9) [6]. In the same fashion, Low [7] reported low factor loadings for items 8 and 9 in the PG construct, and described PG as better explained by two subscales in which PG-Self represented the pleasure or eudaimonic aspect of growing old (items 1 and 2), and PG-Generativity indicated a more altruistic aspect of growing old by setting a good example for others (items 8 and 9). Moreover, Low [7] tested a four-factor model by considering PG-Self and PG-Generativity as two different factors, but although the factor loadings for items 8 and 9 improved, the data did not significantly better fit a four-factor model compared to the original three-factor model. Moreover, items 8 and 9 also seem to highly correlate with each other as compared to items 1 and 2 [11], suggesting these two items might reflect a subgroup of items in the PG scale.

While the full Attitudes to Ageing Questionnaire (AAQ) has recently been translated and validated in Sinhala for use among institutionalized older adults in Sri Lanka [13], to the best of our knowledge, the short form (AAQ-SF) has not yet been validated in this context. The AAQ-SF offers the advantage of reduced participant burden, which is particularly important when working with older adults in residential care. Moreover, while most research has used community samples, this study explored its validity employing elders living in residential homes. Establishing its psychometric properties in this context is crucial to ensure that the measure captures culturally relevant attitudes toward aging and can be applied confidently in research and practice to support healthy aging among Sri Lankan older adults living in retirement homes. This paper explored the construct validity of the AAQ-SF as well as its association with quality of life, gender, and parental status.

## 2. Method

### 2.1. Participants

Inclusion criteria required participants to be aged 55 years or older able to communicate in Sinhala or English, and cognitively capable of completing the study measures. A total of 317 participants were recruited across 13 retirement homes in Sri Lanka using purposive sampling. The age ranged from 55 to 94 years (*M* = 73.6, *SD* = 9.01), and the duration of stay in the retirement homes ranged from 1 to 324 months (*M* = 52.42, *SD* = 55.28). The demographic characteristics of the sample are shown in Table 2.

### 2.2. Translation Procedures

All study materials, including the participant information sheet, informed consent forms, and questionnaires were translated from English to Sinhala using a forward–backward translation method following the guidelines outlined by Guillemin [14] to ensure conceptual and semantic equivalence. First, the first author translated the instruments from English to Sinhala. Then, a government-accredited translator independently back-translated the Sinhala versions into English to verify accuracy and conceptual consistency. Any discrepancies between the original and back-translated versions were reviewed and resolved to produce the final Sinhala versions used in this study. In total, 13 participants completed the English version of the questionnaires, whereas 304 participants completed the Sinhala version, their mother tongue. For the analyses, responses from both language versions were pooled because the questionnaires contained identical items and response formats and the translation procedure ensured conceptual equivalence across the Sinhala and English versions.

### 2.3. Measures

#### 2.3.1. Demographic Questionnaire

A demographic questionnaire was developed for this study to collect information on the participants’ age, gender, ethnicity, educational level, employment status, annual household income, marital status, number of children, duration of residence in the retirement home, and medical conditions.

#### 2.3.2. Attitudes to Ageing-Short Form (AAQ-SF; Laidlaw [6])

The AAQ-SF was used to measure attitudes towards aging. The instrument consists of 12 items and provides scores for three factors: psychological growth (PG, items 1, 2, 8, 9), physical change (PC, items 4, 6, 11, 12), and psychosocial loss (PL, items 3, 5, 7, 10, which are reverse coded). This measurement employs a 5-point Likert scale, and each factor score ranges from 4 to 20. Higher total scores reflect a greater level of positive attitudes towards aging.

#### 2.3.3. Brief Older People’s Quality of Life Questionnaire (OPQOL-Brief; Bowling [15])

This questionnaire measures the perspectives of older adults in relation to their quality of life. It includes a single item assessing global quality of life and 13 items assessing different aspects of quality of life. For the purposes of the current study the results from the global quality of life item were used. Respondents rated the item on a five-point scale from 1 (very good) to 5 (very bad), but responses were reverse coded for data analysis so that higher scores represented higher global quality of life.

### 2.4. Procedure

All procedures were conducted in accordance with the ethical standards outlined by the University’s Human Research Ethics Committee. Written permission was also obtained from the relevant government authority (National Secretariat for Elders) and the administrative authorities (Directors/Managers) of each participating retirement home prior to data collection. Recruitment was carried out between 21 January 2025 and 9 June 2025.

Before data collection, the first author informally assessed each participant’s cognitive and communicative ability to ensure that they could meaningfully engage in the study. This involved asking simple questions about daily activities and observing nonverbal behaviors such as eye contact, facial expressions, and attentiveness. Participants then completed the demographic questionnaire, providing factual responses (e.g., length of residence and medical conditions). Only individuals who demonstrated adequate understanding and communication during this screening proceeded to the research measures, while those who were unable to respond appropriately were not invited to participate further. The researcher then provided participants with a copy of the questionnaire, read each item aloud, and recorded participants’ responses. To minimize potential social desirability or interviewer effects, the researcher followed a standardized script, read each question exactly as written, and maintained a neutral tone, posture, and facial expression throughout administration.

### 2.5. Data Analysis

Construct validity was tested using CFA in IBM^®^ SPSS^®^ AMOS (version 29, IBM Corp., Armonk, NY, USA). Model fit was assessed using multiple indices, including χ^2^, χ^2^/df, the Tucker–Lewis index (TLI), the comparative fit index (CFI), and the root mean square error of approximation (RMSEA). The following cut-off values were considered acceptable: χ^2^/df < 3, TLI and CFI ≥ 0.90, RMSEA ≤ 0.06 [16]. The Akaike’s information criterion (AIC) was reported to compare different models, and the smaller the values are, the more fit is considered [17]. Standardized loading estimates ≥0.50 were considered appropriate [18]. Internal consistency was assessed using Cronbach’s alpha values (appropriate values ≥ 0.70 [18]). To explore the convergent validity of the measure, correlation coefficients were used to explore associations previously found in the literature between the subscales of the AAQ-SF and quality of life.

Attitudes to aging was also compared for males and females using an independent *t*-test. The previous literature (e.g., [7]) indicated no differences between males and females. We also predicted no differences in attitudes to aging for those who had or did not have children. It is noted that the previous literature had revealed more negative attitudes towards aging for those with poor health as compared to those with good health. We did not compare people with and without chronic medical conditions because the majority (85%) of our sample had a common age-related medical condition.

## 3. Results

Table 3 shows the bivariate correlations between the items of the AAQ-SF. The table shows nonsignificant correlations among two subgroups of items of the PG subscale. Specifically, items 1 and 2 (PG-Self) did not correlate significantly with items 8 and 9 (PG-Generativity), suggesting the items may not capture the underlying concept of psychological growth in the same manner. Moreover, items 8 and 9 (PG- Generativity) did not have any significant correlations with items in the PL factor.

### 3.1. Confirmatory Factor Analysis (CFA)

The original three-factor model (model 1) was tested using CFA maximum likelihood estimation. Examination of the output revealed no missing values, no univariate or multivariate outliers, appropriate univariate normality (skewness values ranged between −1.06 and 0.78; kurtosis values ranged between −1.67 and −0.45), but the presence of multivariate nonnormality (kurtosis = 20,84; critical ratio = 10.12), and a poor fit (Table 4).

Standardized regression loadings were relatively appropriate with most values around 0.50. However, item 12 (I keep myself as fit and active as possible by exercising) and item 4 (I don’t feel old), both from the PC subscale, had low loadings of 0.24 and 0.31, respectively. Importantly, the factor loadings for three out of the four items of the PG factor seemed problematic—items 8 (It is very important to pass on benefits of my experiences to younger people) and 9 (I want to give a good example to younger people) were close to 0 in the PG scale, and the standardized regression loading of item 2 was 1.21, suggesting issues with the PG subscale overall. Modification indices suggested a covariance between error for items 8 and 9.

Following Byrne’s [17] recommendations, a covariance was added between the errors of items 8 and 9, and asymptotic distribution-free estimation was used to address multivariate nonnormality. The results of this model (model 2) showed a good fit (Table 4). However, the standardized regression loadings for items 2, 8, and 9 showed the previous limitations as in model 1, and the factor loadings for items 4 and 12 were 0.30. Heywood cases were observed. Specifically, the standardized factor loadings for items 2, 8, and 9 were inadequate (1.24, −0.06, and −0.06, respectively), and a negative error variance for item 2 was found (−0.70). The psychometric stability of the PG factor seemed compromised.

Given the fact that PG has been deemed as a multidimensional factor [6,7], a third model was tested in which PG was divided into two subscales and named as in Low’s [7] article: PG-Self (consisting in items 1 [It is a privilege to grow old] and 2 [There are many pleasant things about growing older]) and PG-Generativity (items 8 [It is very important to pass on benefits of my experiences to younger people] and 9 [I want to give a good example to younger people]). A four-factor model was tested (model 3) and showed good fit (Table 4). The standardized regression loadings showed an improved and adequate fit, except items 12 and 4 (both from the PC subscale), which loadings were 0.31 and 0.32, respectively (low but possible and acceptable values), and item 2 (PG), which loading was 1.22, suggesting that issues with the PG structure were not totally solved. Specifically, the standardized factor loadings for items 2, 8 and 9 were extremely high (1.22, 0.99, and 1); negative error variances (e2 = −0.62, e9 = −0.01), as well as a negative residual covariance between items 8 and 9 (−0.07) were found. The results reinforced the instability of the PG scale. A comparison of the factor loadings for the three models is presented in the Appendix A, Table A1.

### 3.2. Internal Reliability and Convergent Validity

The results showed suboptimal internal reliability (Cronbach’s αs ≤ 0.70) for the three subscales of the three-factor model of PG (α = 0.58), PC (α = 0.56), and PL (α = 0.65). When PG was split into two factors, there was high internal reliability for PG-Self (α = 0.79) and PG-Generativity (α = 0.96).

On the item assessing global quality of life from the OPQOL-brief, 10 (3%) participants reported very bad quality of life, 34 (11%) bad, 110 (35%) alright, 132 (42%) good, and 31 (10%) very good. The mean score on the five-point scale was 3.44 (*SD* = 0.92).

The AAQ-SF showed good convergent validity with quality of life. Positive attitude to aging was associated with higher quality of life for PG (*r* = 0.25, *p* < 0.001), PC (*r* = 0.43, *p* < 0.001), and PL (*r* = 0.41, *p* < 0.001).

Contrary to what we predicted, men (*M* = 11.03, *SD* = 3.43) reported more psychological growth than women (*M* = 9.98, *SD* = 3.37), *t*(315) = 2.65, *p* = 0.008, specifically about the benefits of growing old (PG-Self), but no statistically significant differences between men and women were found for PC (*M*_men_ = 12.49, *SD*_men_ = 3.79; *M*_women_ = 12.04, *SD*_women_ = 3.79) and PL (*M*_men_ = 11.32, *SD*_men_ = 3.60; *M*_women_ = 10.60, *SD*_women_ = 3.59), both *p*s > 0.05. As predicted, having or not having children did not affect attitudes towards aging: PG (*M*_no_ = 10.56, *SD*_no_ = 3.33; *M*_yes_ = 12.12, *SD*_yes_ = 3.53), PC (*M*_no_ = 10.98, *SD*_no_ = 3.36; *M*_yes_ = 10.72, *SD*_yes_ = 3.88), or PL (*M*_no_ = 12.41, *SD*_no_ = 3.73; *M*_yes_ = 11.97, *SD*_yes_ = 3.86), all *p*s > 0.05.

## 4. Discussion

The present study examined the construct validity of the AAQ-SF and its associations with quality of life, gender, and parental status among 317 participants residing in 13 retirement homes in Sri Lanka. This first attempt of validation of the AAQ-SF provides an important contribution for researchers interested in studying the aging population within the Sri Lankan context. Evidence for construct validity was established through correlation analyses and CFA, although the consistency of the PG scale might require further investigation.

In the present study, the correlation analyses revealed nonsignificant associations between the two subgroups of items within the PG subscale. Specifically, the PG-Self items (items 1 and 2) did not correlate with the PG-Generativity items (items 8 and 9), suggesting that the four items may not systematically capture the same meaning associated with the construct of psychological growth. The current results are consistent with the idea that PG might consist of two aspects of growth, one related to the benefits of growing old and the other about the opportunity to share knowledge with younger generations, as mentioned by the original authors of the measure [6,7]. Consistent with the multidimensionality of the PG factor, the CFA suggested that the best fit corresponded to a four-factor model wherein PG was divided between PG-Self and PG-Generativity. The four-factor model made items 8 and 9 correlate with the new PG-Generativity, items which were not correlated strongly with PG in the three-factor model, similarly to the results obtained by Low [7]. However, it is important to note that although the fit indices (e.g., TLI, CFI, RMSEA) showed adequate fit, Heywood cases suggested misspecification for the PG-Self and PG-Generativity (as well as for the PG), such as extremely high factor loadings in the PG-Generativity scale, suggesting item redundancy. Future studies may consider reanalyzing the data removing item 2 and/or items 8 or 9. Laidlaw [6], in their initial exploratory factor analyses, also found that PG captured “qualitatively distinct aspects of attitudes and experiences related toward aging” (p. 117), and that items 1 and 2 (PG-Self) overlapped with the PC scale, but decided that removing items 1 and 2 would limit the construct, mentioning that the “PG is nuanced construct comprising different elements; thus no scale can aim to provide a comprehensive coverage of this” (p. 120).

The CFA indices showed that a three-factor model (i.e., PG, PC, and PL) was as psychometrically sound as the four-factor model, and our CFA results for the three-factor model showed similar fit values compared to the previous literature [6,7,11]. Similar to previous studies [11], we also found a high correlation between items 8 and 9 and low factor loadings for item 12 (PC; keeping fit). This is relevant because the current study employed residents of elders’ homes, while previous validation studies have used community-dwelling samples.

The internal consistency of the AAQ-SF was suboptimal, with Cronbach’s alpha values below 0.70 (except when PG was divided into two subscales). However, references [6,7] also reported relatively low internal reliability, particularly for the PG subscale, suggesting that the items within this factor may not fully capture a single underlying construct. Further, the low number of items per subscale may also have negatively impacted the internal consistency values.

In addition to construct validity, the present findings provide convergent validity evidence for the AAQ-SF. Positive attitudes toward aging, particularly in relation to physical change and psychosocial loss, were linked with greater quality of life. These results align with previous research showing that older adult’s perceptions of their own aging processes can significantly influence their health outcomes [19]. Moreover, having a greater sense of purpose in life and higher levels of personal growth have been found to be strongly associated with better quality of life among older adults [20]. Aging is associated with new life challenges; some older adults experience this period as a time of growth and personal discovery, such as learning new skills, whereas others perceive it as a time marked by losses of loved ones and psychosocial decline [21]. Overall, the findings of the present study indicate that psychosocial loss, physical change, and psychological growth collectively influence the quality of life of older adults.

Contrary to expectations, the current study found that men reported higher levels of psychological growth than women, particularly regarding the perceived benefits of growing older (PG-Self). It is unclear why men scored higher than women when reporting growing old as pleasant or as a privilege. However, this finding may be related to structural and demographic factors. Life expectancy is generally shorter for men than for women and therefore reaching older age may be perceived as a privilege among men. In addition, gender inequalities across the life course, particularly in employment opportunities and income, may shape experiences in later life. Due to gender segregation in the labor market and less stable employment histories, women are often less likely to be covered by pension schemes and may have fewer financial resources in older age. At the same time, men may retain certain forms of social status associated with previous occupational roles and economic security, which could influence how aging is perceived [22]. No significant gender differences were observed for the physical change and psychosocial loss subscales. Reference [7] also found negligible gender-based differences across all AAQ-SF subscales. As expected, parenthood did not influence any of the positive aging indices, including psychological growth, physical change, or psychosocial loss.

In the current study, participants were older adults residing in 13 retirement homes in Sri Lanka, and this may limit the generalizability of the findings to the community-dwelling or nursing home populations of older adults in Sri Lanka. Moreover, the CFA revealed possible multicollinearity among items 8 and 9, suggesting that the meaning of these two items might have been interpreted as very similar by the participants, as well as possible overspecification for the PG factor and its subscales.

## 5. Conclusions

Future studies in Sri Lanka with different samples are needed to further support the validity of the AAQ-SF in Sri Lanka. Nevertheless, the findings of the present study provide preliminary evidence supporting the construct validity and the convergent validity of the AAQ-SF among older adults in Sri Lanka. However, given the observed limitations in some psychometric indicators, the results should be interpreted with caution and further validation research is warranted.

## Figures and Tables

**Table 1:** CFA and internal reliability values for a 3-factor model reported in previous research.

Authors	CFA				Internal Reliability		
	TLI	GFI	CFI	RMSEA	PG	PC	PL
Laidlaw [6]	0.83–0.84	-	0.87–0.88	0.08	0.61–0.62	0.72–0.73	0.68–0.72
Low [7]	-	-	0.87	0.05	0.61	0.78	0.71
Khongsirisombat [11]	-	0.96	0.98	0.04	0.76	0.76	0.70

Note. CFA = Confirmatory Factor Analysis, TLI = Tucker–Lewis index, GFI = Goodness-of-fit index, CFI = Comparative fit index, RMSEA = Root mean square error of approximation. PG = Psychological growth, PC = Physical change, PL = Psychosocial loss.

**Table 2:** Characteristics of the participants (*N* = 317).

Characteristics	*N*	%
Sex		
Male	115	36.3
Female	202	63.7
Ethnicity		
Sinhala	295	93.1
Other	22	6.9
Marital status		
Never married	112	35.3
Married or domestic partnership	31	9.8
Separated or divorced	44	13.9
Widowed	130	41.0
Education		
No formal education	72	22.7
Primary school	90	28.4
Secondary school	140	44.2
Diploma	9	2.8
Bachelor’s degree	5	1.6
Postgraduate degree	1	0.3
Income (Rs.) ^1^		
No income	276	87.1
Less than 20,000 LKR.	25	7.9
20,000–39,999 LKR.	9	2.8
40,000–59,999 LKR.	6	1.9
60,000–79,999 LKR.	1	0.3
Medical conditions ^2^		
No	49	15.5
Yes	268	84.5

Note. ^1^ LKR = Sri Lankan rupee. Exchange rate on 6 November 2025: 1 USD = 308.25 LKR. ^2^ The most common health issues reported were blood pressure, diabetes, cholesterol, and heart disease.

**Table 3:** Item correlations of the AAQ-SF.

Items	1	2	8	9
Psychological growth (PG)				
PG-Self				
1. It is a privilege to grow old	-			
2. There are many pleasant things about growing older	0.65 **			
PG-Generativity				
8. It is very important to pass on the benefit of my experiences to younger people	−0.04	−0.01	-	
9. I want to give a good example to younger people	−0.03	−0.02	0.93 **	-
Physical change (PC)				
4. I do not feel old	−0.01	0.02	0.10	0.13 *
6. I have more energy now than I expected for my age	0.15 *	0.27 **	0.16 *	0.15 *
11. My health is better than expected for my age	0.04	0.16 *	0.20 **	0.16 *
12. I keep myself as fit and active as possible by exercising	0.10	0.13 *	0.09	0.13 *
Psychosocial loss (PL)				
3. Old age is depressing time of life	0.15 *	0.28 **	0.03	0.06
5. I see old age mainly as a time of loss	0.07	0.28 **	0.04	0.05
7. As I get older, I find it more difficult to make new friends	0.12 *	0.15 *	0.03	0.08
10. I feel excluded from things because of my age	0.08	0.16 *	0.04	0.05

Note. * *p* < 0.05, ** *p* < 0.001.

**Table 4:** Confirmatory factor analysis fit statistics for the AAQ-SF.

Model	χ^2^(df)	χ^2^/df	TLI	CFI	RMSEA	AIC
Model 1	720.57(51) *	14.13	0.31	0.46	0.20	774.57
(3 factors)						
Model 2	95.31(50) *	1.91	0.94	0.95	0.05	151.31
(3 factors with error covariances between items 8 and 9)						
Model 3	79.30(48) *	1.65	0.96	0.97	0.05	139.30
(4 factors with PG divided)						

Note. * *p* < 0.05. Model 1 and 2 (3 factors) refer to PG (psychological growth), PC (physical change), and PL (psychosocial loss). Model 3 (4 factors) refer to PG-Self, PG-Generativity, PC and PL.

## Data Availability

Data is available from the corresponding author.

## Appendix A

**Table A1:** Standardized Factor Loadings for the three models.

Factors/Items	Model 1	Model 2	Model 3
PG			
1	0.54	0.54	0.55
2	1.21	1.24	1.22
8	−0.00	−0.06	0.99
9	−0.03	−0.06	1.00
PC			
4	0.31	0.29	0.32
6	0.82	0.87	0.86
11	0.71	0.75	0.79
12	0.24	0.30	0.31
PL			
3	0.58	0.61	0.63
5	0.63	0.68	0.67
7	0.46	0.45	0.45
10	0.62	0.63	0.64

Note. Model 1 (3 factors) and Model 2 (3 factors with covariance between errors items 8 and 9) have the following factors PG (psychological growth), PC (physical change), and PL (psychosocial loss). Model 3 has four factors, wherein PG is split into PG-Self, PG-Generativity, and the other two are PC and PL.
